# Remote Management of Patients after Total Joint Arthroplasty via a Web-Based Registry during the COVID-19 Pandemic

**DOI:** 10.3390/healthcare9101296

**Published:** 2021-09-29

**Authors:** Michele Ulivi, Luca Orlandini, Valentina Meroni, Mario D’Errico, Arianna Fontana, Marco Viganò, Laura Mangiavini, Roberto D’Anchise, Franco Parente, Roberto Pozzoni, Valerio Sansone, Luigi Zagra, Giuseppe M. Peretti

**Affiliations:** 1 IRCCS Istituto Ortopedico Galeazzi, Via Riccardo Galeazzi 4, 20161 Milan, Italy; micheleulivi@msn.com (M.U.); orlandini_luca@yahoo.com (L.O.); valedoc.meroni@gmail.com (V.M.); marioderric@gmail.com (M.D.); arianna.fontana@grupposandonato.it (A.F.); laura.mangiavini@unimi.it (L.M.); roberto.danchise@danchise.it (R.D.); rpozzoni@tiscali.it (R.P.); valerio.sansone@unimi.it (V.S.); luigi.zagra@fastwebnet.it (L.Z.); giuseppe.peretti@unimi.it (G.M.P.); 2 Department of Biomedical Sciences for Health, Università degli Studi di Milano, 20133 Milan, Italy; 3 IRCCS Istituto Ortopedico Galeazzi, Via Monreale 18, 20148 Milan, Italy; franco.parente1@tin.it; 4 Department of Orthopaedics, Universita’ degli Studi di Milano, 20161 Milan, Italy

**Keywords:** hip arthroplasty, knee arthroplasty, registry, remote monitoring, COVID-19

## Abstract

Background: In 2020, due to the outbreak of the COVID-19 (Coronavirus Disease 2019) pandemic, patients who underwent total joint arthroplasty were not able to undergo the proper postoperative surgical and rehabilitative care. This study aims to evaluate the potential of a web-cloud-based database on patients’ follow-up in extraordinary situations, when a traditional in-person follow-up cannot be warranted. Methods: Patients who underwent joint arthroplasty at our Institute between 21 February and 16 March 2020 were included in the study group and were matched to a similar population undergoing joint arthroplasty in February/March 2019. All patients routinely complete questionnaires before and after treatment, including patient-reported outcome measures such as the Visual Analogues Scale (VAS), Knee/Hip Injury and Osteoarthritis Outcome Score Physical Function Short Form (KOOS-PS/HOOS-PS) and Short-Form Health Survey (SF-12) for the monitoring of clinical improvements. Results: 56 (study group) and 144 (control group) patients were included in the study. Both groups demonstrated significant improvements at 3 months. HOOS-PS improvement was significantly reduced in the 2020 group compared to 2019 (21.7 vs. 33.9, *p* < 0.001). This reduction was related to intense physical activities. Similarly, the functional score improvement related to these activities was reduced for patients undergoing knee replacement (8 vs. 10, *p* < 0.05). Conclusions: The web-based Institute Registry emerged as a meaningful and sensitive tool during an extraordinary situation such as the COVID-19 pandemic to monitor patients’ progression after total joint arthroplasties. Thanks to this tool, it was possible to observe that the prevention of usual postoperative care due to pandemic-related restrictions did not alter the benefits observed after joint replacement surgeries, even if this condition reduced the postoperative improvements in the most burdensome physical activities. A broader use of this kind of tool would improve and potentially reduce the burden and costs of postoperative patients’ monitoring in standard and extraordinary conditions. In addition, the systematic remote collection of data would allow for the identification of relevant differences in clinical outcomes in specific conditions or following the modification of treatment and rehabilitation protocols.

## 1. Introduction

The electronic web-based registry “Registro Protesi H&K” was established at the Istituto Ortopedico Galeazzi in 2016 for the monitoring of patients undergoing joint arthroplasty. It collects information related to surgical interventions, diagnostic imaging (e.g., X-rays, CT and RMN, or any other imaging technique) as well as the type of implanted devices. Moreover, it stores demographic data and Patient Reported Outcomes Measures (PROMs) including pain intensity assessments and clinical scales, collected both pre- and postoperatively.

On 11 March 2020, the World Health Organization declared the pandemic status as a consequence of the rapid global spread of SARS-CoV-2 [1]. Italy, and in particular the Lombardy region, was heavily impacted [2,3]. In order to reduce the spread of the disease, containment measures were adopted which included a complete lockdown [4,5,6]. The healthcare system organization was consequently modified with a drastic reduction in orthopedic surgical procedures and a complete halt to elective surgeries. This status lasted from 16 March to 3 June 2020 at our Institute. This caused a reduction in the number of hip and knee implants [7] in parallel with the worsening of the spread of the COVID-19 epidemic in Italy [8]. The healthcare activity suspension also prevented regular postoperative care and rehabilitation activities usually prescribed to patients after total join arthroplasties (TJA).

The main purpose of this study is the evaluation of the remote monitoring of patients using a web-based registry as a tool to record patients’ progression after TJA. The hypothesis is that in the presence of extraordinary situations, such as the total lockdown caused by the COVID-19 pandemic, these tools would represent reliable sources of information related to the postoperative recovery of patients. To test the potential of the registry in identifying possible differences between pandemic and pre-pandemic postoperative progression, the short-term results of patients who underwent TJA in the immediate period before the COVID-19-related lockdown were compared to those of patients who underwent the same procedure in March/April 2019.

## 2. Materials and Methods

### 2.1. Study Design

**Figure 1:**
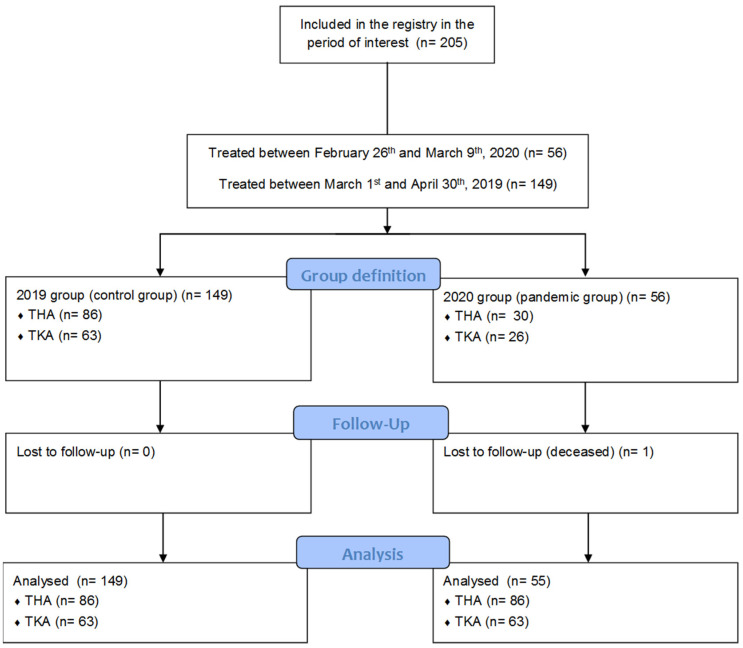
Diagram of patients’ inclusion and follow-up during the study. THA: total hip arthroplasty; TKA: total knee arthroplasty.

The present study is a retrospective cohort investigation, comparing patients who underwent Total Joint Arthroplasty in the immediate days before the application of the lockdown due to the COVID-19 pandemic in 2020 with a historical cohort of patients who underwent these procedures in the same season in 2019 (Figure 1).

### 2.2. Patients and Data Collection

All patients referring to 7 Institute’s Units and undergoing hip or knee replacement at the IRCCS Istituto Ortopedico Galeazzi are included in the Institutional Registry (H&K Datareg Register). These 7 orthopedic Units are actively contributing to the Institutional Registry, which is a single collector of information related to surgical operations, such as the type of implanted device, diagnostic imaging (e.g., X-rays, CT and RMN, as well as any other imaging technique) collected pre- and postoperatively as well as anonymized data about the patient demographics (age, gender, Body Mass Index, diagnosis). It also stores Patient Reported Outcomes Measures (PROMs) and clinical scales such as the Harris Hip Score (HHS) [9] and the Knee Society Score (KSS) [10]. The clinical scales are administered by a qualified healthcare professional, i.e., the resident, at baseline and follow-ups, which are routinely scheduled at 3, 6, 12 months and subsequently at 2, 5 and 10 years after surgery [11]. Regarding PROMs, all patients at the same previously mentioned follow-ups completed the Visual Analogue Scale for Pain (VAS) and the 12-item Short Form (SF-12). In addition, the Hip Disability and Osteoarthritis Outcome Score (HOOS-PS) and Knee injury and Osteoarthritis Outcome Score (KOOS-PS) are completed for hip and knee patients, respectively. The satisfaction rate for the patient was also collected. Since these latter PROMs are intended to be representative of the patients’ voices without doctor mediation, they are usually collected before the visit. An automatic alert was generated for those patients providing an e-mail address; if this was not possible (e.g., the elderly), completion of the PROMs took place with the support of IOG operators. They helped patients in loco, providing tablets with digital forms and supporting the completion of the tests, or they interviewed patients through a phone call.

In the present study, due to COVID-19 pandemic constraints and the consequent impossibility of conducting physical follow-ups on patients, only the 3-month telephone follow-up visit was considered for analysis, in line with the study hypothesis. PROMs include the Italian-validated version of the SF-12 (mental scale, MCS and physical scale, PCS), the VAS and the short form of the Hip Disability and Osteoarthritis Outcome Score (HOOS-PS) and Knee Injury and Osteoarthritis Outcome Score short forms (KOOS-PS) [12,13,14,15]. The SF-12 MCS and PCS are useful tools for the identification of mental and physical health, respectively, ranging 0–100 with higher scores implying better conditions. The VAS is a validated instrument for grading pain, and it is a continuous scale ranging 0–10 with higher scores indicating higher pain intensity. Similarly, higher scores on the KOOS-PS and HOOS-PS identify the worst conditions related to a patient’s ability to perform physical activities.

The follow-ups were performed by phone calls, and the patients were asked to rate their satisfaction on a 5-point scale (0 = very dissatisfied; 1 = somewhat dissatisfied; 2 = good; 3 = somewhat satisfied; 4 = excellent). Informed consent for data collection was obtained from all patients, according to the protocol approved by the Institutional Review Board (IRB).

Inclusion criteria for the pandemic group (2020) were: age >18 years old, a total hip arthroplasty (THA) or a total knee arthroplasty (TKA) procedure performed between 21 February and 16 March 2020. These dates correspond with the day of the first COVID-19 case identified in Italy [16] and the day of the last surgery performed before the lockdown, resulting in the suspension of elective surgical activity. Inclusion criteria for the control group (2019) were: age >18 years old and TKA or THA performed between 1 February and 31 March 2019. Exclusion criteria were revision surgeries and the impossibility/refusal to sign the informed consent.

### 2.3. Procedures

Patients in the 2019 comparative group underwent standard rehabilitation programs for THA and TKA in the different Units composed of early (day 0–1) mobilization. From day 0–1 and from day 2 onwards, the following were considered: recovery of postural steps, articular range of motion (ROM), muscular tone, introduction to upright position, education to walk with crutches in full weight-bearing, avoidance of prolonged immobilization. This program, considering intra-patient differences, continued with home-based exercises after dismissal, followed by a complete patient compliance check. The program consisted of exercises at home under the supervision of a physical therapist 3 times a week or through hospitalization for at least 3 weeks in an appropriate rehabilitation facility. The control of the patient’s compliance by the surgeon was carried out 2 weeks after surgery and, if necessary, with intermediate controls until the first follow-up control scheduled at 3 months after surgery [17,18].

Patients in the 2020 group could not be followed directly after a short-time-frame discharge. They did similar immediate postoperative protocols, but then they were instructed to conduct rehabilitation programs on their own without medical guidance or an overview.

Medical needs including the assessment of surgical wound healing were managed at home by the family physician.

### 2.4. Statistical Analysis

The data analysis was performed using R software v3.6.3 (R Core Team, Wien, Austria). The Shapiro–Wilk test was used to assess the data distribution. In the case of normal distribution, data were compared using the unpaired Student’s t-test; otherwise, the Mann–Whitney test was applied. In accordance with the data distribution, the median and ranges (or interquartile range, IQR) are reported throughout the manuscript. An analysis of covariance (ANCOVA) approach was applied to test the between-group differences adjusted for the different baseline values. Percentage changes at 3 months were calculated as: (value at 3 months − value at baseline)/value at baseline. Categorical variables were compared between the two groups using Fisher’s exact test. *p* values < 0.05 were considered statistically significant. Effect sizes were calculated using Cohen’s d [19].

## 3. Results

### 3.1. Patients Demographics

A total of 56 patients were operated on in the considered period of 2020, while 149 underwent total joint arthroplasty (TJA) in February and March 2019. One patient died in 2020 before the 3-month follow-up, for reasons unrelated to COVID-19; therefore, 55 patients were included in the 2020 group for the analyses. The death rate did not differ between the two groups (*p* = 0.269). Concerning THA, 30 patients were operated on in 2020, while 86 underwent surgery in 2019. Regarding the group receiving TKA, 25 were operated on in 2020 and 63 in 2019. No patients refused to answer the follow-up questionnaires at 3 months, and thus the response rate was 100% in 2019 and 98.2% in 2020. The groups were comparable in terms of diagnosis, age, gender and BMI. Table 1 summarizes these data.

### 3.2. Preoperative and 3-Month PROMs in THA Patients

**Figure 2:**
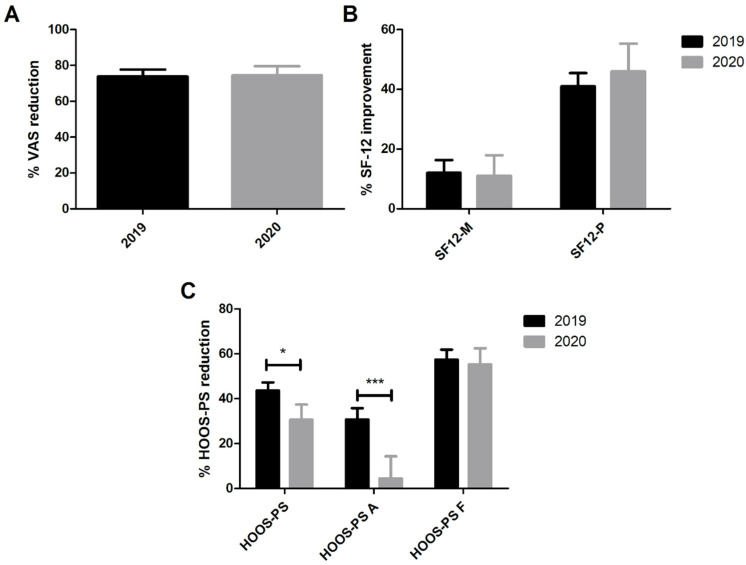
VAS (**A**), SF-12 (**B**) and HOOS-PS (**C**) percentage improvements in patients treated for THA in 2019 and 2020. * *p* < 0.05, *** *p* < 0.001. Data expressed as mean ± SEM.

Patients who underwent THA in 2020 demonstrated higher preoperative VAS and HOOS-PS scores compared to those who underwent this procedure in 2019, thus suggesting the presence of more acute symptomatology in 2020. No differences were observed in mental and physical SF-12 preoperative scores. After 3 months, the VAS score was similar in the two groups, while HOOS-PS scores were still significantly higher in the 2020 group. Indeed, both scores significantly improved compared to the baseline in each group (*p* < 0.001), as well as with the physical SF-12 (Figure 2A,B). On the contrary, the mental SF-12 did not show any improvement between the baseline and 3-month evaluations. Table 2 reports these findings. Considering the fold change in HOOS-PS scores, patients operated on in 2019 demonstrated greater improvements compared to those operated on in 2020 (Figure 2C). In fact, the median reduction was −51.6% (IQR: −66.3%, −28.6%) in 2019, while it resulted in a reduction of −26.5% (IQR: −46.0%, −10.0%) in 2020 (*p* = 0.031). In addition, the effect size (Cohen’s d) of the HOOS-PS improvement in 2019 was 1.55, and in 2020 it was 0.97. Interestingly, this difference was due to a smaller increase in the HOOS-PS Activity subscale (*p* < 0.001), while the HOOS-PS Functional subscale did not demonstrate any difference between the study groups (*p* = 0.765). Average satisfaction was good or excellent in both groups, without a significant difference between the pandemic and the control group.

### 3.3. Preoperative and 3-Month PROMs in TKA Patients

**Figure 3:**
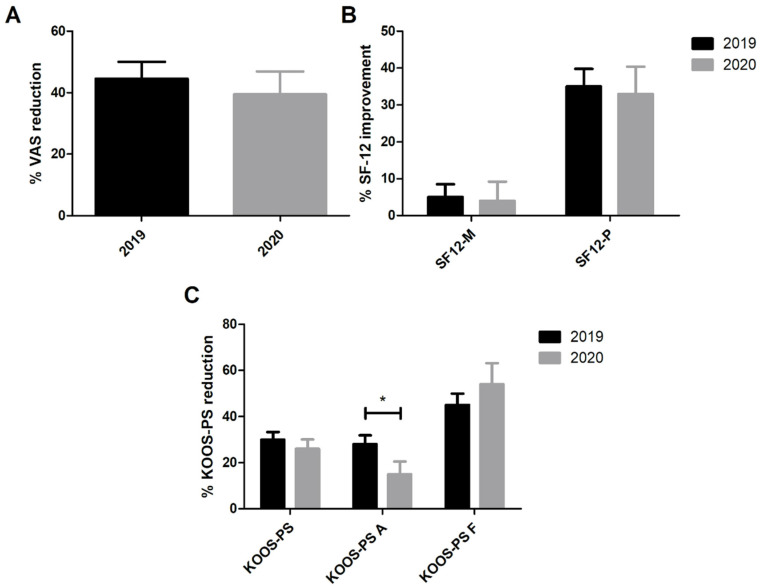
VAS (**A**), SF-12 (**B**) and KOOS-PS (**C**) percentage improvements in patients treated for TKA in 2019 and 2020. * *p* < 0.05. Data expressed as mean ± SEM.

No differences were observed between groups in any of the PROMs at preoperative or 3-month evaluations concerning patients who underwent TKA. Again, significant differences were observed between preoperative and 3-months scores within the same group concerning the VAS, physical SF-12 and KOOS-PS, but not the mental SF-12 (Figure 3). Table 3 summarizes these results. Similar effect sizes were observed in the 2019 and 2020 cohorts, with a Cohen’s d of 1.19 and 1.06, respectively. Interestingly, considering the Activity subscale of the KOOS-PS, a significant difference was found between the groups in favor of 2019 over 2020 (*p* = 0.015). The mean reduction in this parameter was 28% in the 2019 group and 15% in the 2020 group. Patients’ satisfaction appeared superior in 2020 compared to 2019 even if this difference was not statistically significant (*p* = 0.078).

## 4. Discussion

The aim of this study is the evaluation of the Institutional Registry for joint arthroplasties as a tool for remote monitoring patients’ progression after these types of surgeries.

In our study, we demonstrated that in spite of the occurrence of an unpredicted and sudden condition leading to a complete nationwide lockdown such as the occurrence of the COVID-19 pandemic, we have been able, thanks to the Registry, to offer a reduced follow-up involving all the patients who underwent THA and TKA in the period going from the outbreak of the pandemic to the complete standstill of elective surgical interventions. The analysis and evaluation of the self-administered PROMs confirmed that the VAS, SF-12 PCS, HOOS-PS and KOOS-PS demonstrated significant improvements in both groups when compared to baseline pre-operative values, with values clearly above MCIDs [9,20,21]. These improvements were comparable in magnitude with previous studies for both THA and TKA, which showed improvements at 3 months with effects sizes 2.20 and 1.15 on the HOOS-PS and KOOS-PS, respectively [22,23], while in our study, these effects were 1.55 and 1.19 in 2019 and 1.06 and 0.97 in 2020, for the HOOS-PS and KOOS-PS, respectively. Despite the slight differences in studies and cohorts, all the observed effects sizes are considered “large” [19]. Notably, these studies also reported an association between PROMs and functional tests, supporting the idea that these tools would be representative not only of the perceived pain/disability but also of the patients’ real functional improvements [22,23].

The overall response rate in our series of patients was 99.5%. This value is extremely high, in particular when compared to other registry-based reports [24]. The reason for this unexpected response rate is due both to the short time period from a major surgery as well as to the activity of qualified dedicated personnel who directly contacted patients and checked the completion of the questionnaires.

The unusual and extraordinary conditions imposed by the COVID-19 pandemic did not alter the overall clinical importance of total joint replacement even in the absence of a monitored rehabilitation program. Patients’ satisfaction in relation to the procedure was at least “good” in all groups. In addition, thanks to the Registry, it was possible to identify a reduced improvement in terms of the ability to perform specific tasks with respect to patients who underwent the same procedure in 2019. These tasks were identified in the HOOS-PS and KOOS-PS Activity subscales, corresponding to items 2 and 3, respectively, that are related to burdensome physical activities [25], as summarized in Table 4.

Due to the aforementioned issues, the 2020 group underwent only one surgical follow-up (day 14) after discharge. Then, these patients did not complete a proper standardized rehabilitation protocol, which usually includes domestic or hospitalized physiotherapy [10,26].

In addition to rehabilitation suspension, the above-mentioned differences noticed in both THA and TKA may be due to the interruption of the interaction between the patient and the surgeon in the first weeks after surgery, when the personal contact with the health care provider may exert a positive effect on the patient’s outcome. In the first days after surgery, a patient may experience some fear of ambulation or in performing the exercises, and the surgeon usually is able to reassure the patient. The limited mobility due to the lockdown could also have played a major role in the reduced recovery, as it was also described to impair the physical conditions of healthy subjects [27].

Telerehabilitation was proposed in recent years as a possible substitute to traditional physical therapy, the impact of which is still controversial [26,27]. In addition, the sudden outbreak of the pandemic did not allow a timely organization of these activities. Moreover, these patients, due to their age, their comorbidities and the absence of feedback with the surgeon, may experience difficulties in autonomously pursuing the rehabilitation program [28].

We did not detect any significant difference between the study groups with respect to the SF12 Mental Score. This finding appears in contrast with the hypothesis that the pandemic may have had a negative effect on the psychological recovery of our patients [29]. A possible explanation is that only highly motivated patients decided to undergo the surgery in this extremely difficult period, whereas less motivated subjects may have decided to postpone the surgery. In addition, it has to be considered that the SF-12 MCS questionnaire improved poorly in both groups, suggesting that the psychological recovery may be small in this category of patients or that this score does not represent an effective method to record these improvements, as previously demonstrated by other authors [21]. Moreover, the SF12 MCS questionnaire has never been tested in similar situations, and, thus, it may not be able to reflect the mental state of patients during a pandemic.

This work suffers from limitations. It relies on a retrospective analysis of prospectively collected data, and the study groups were not matched. Thus, significant differences at baseline were possibly observed due to chance. Nevertheless, the use of an ANCOVA approach, representing the most effective analysis strategy in these cases, allowed for adjusting for these differences. In addition, since it was not possible to assess patients’ compliance to the rehabilitation protocols, the analysis was performed on an intention-to-treat basis. Moreover, the study relies on self-reported questionnaires only, since it was not possible to collect objective data; thus, the observations were not validated by medical professionals. On the other hand, the inclusion in the registry of all patients undergoing arthroplasties at the different Units of the Institute (i.e., treated by different surgeons/equips) allows for a partial generalization of the results obtained. In this context, a continuous and granular analysis of internal-registry-generated data is helpful and sensitive enough not only to understand and predict operational burdens linked to follow-up activities, as we reported in a previous publication [30], but also to detect specific variations in PROMs. Whether these observed differences, though statistically significant, are truly clinically important is not known yet. Further observation in the next years’ follow-ups may reveal if a Minimal Clinically Important Difference (MCID) can be detected in the medium and long term.

Based on our observations, we conclude that the outcomes of THA and TKA elective surgeries, performed during exceptional periods preventing in-person care, are non-inferior compared to the procedures performed when supervision and standard rehabilitation protocols were possible. In fact, there were no substantial differences in short-term outcomes in these procedures during a pandemic period and a normal period concerning the SF12, the VAS score, satisfaction, and the death rate at 3 months after the procedure.

## 5. Conclusions

The availability of a web-based Institute Registry emerged as a meaningful and sensitive enough tool to identify differences in the recovery after joint arthroplasties.

In this study, we observed that an extraordinary situation such as the COVID-19 pandemic did not alter the benefits observed after total joint arthroplasties, while the prevention of rehabilitation and the reduced mobility imposed by the national lockdown reduced the improvements related to the most burdensome physical activities. This study, in our opinion, highlights the importance of the postoperative patient/surgeon relationship and follow-up, rehabilitation protocols and the possibility of moving without restrictions for the achievement of an optimal recovery after joint arthroplasty.

These results are encouraging with respect to the use of registries to improve the understanding of patients’ postoperative needs and possibly to help further research to define relevant Minimal Clinically Important Differences (MCIDs) better. In addition, a broader use of this kind of tool would improve and potentially reduce the burden and costs of postoperative patients’ monitoring in standard and extraordinary conditions. In addition, the systematic remote collection of data would allow for the identification of relevant differences in clinical outcomes in specific conditions or following the modification of treatment and rehabilitation protocols.

## Figures and Tables

**Table 1:** Patients’ Demographics.

	2019	2020	*p* Value
**Overall**			
**Age**	69 (38–97)	69 (44–84)	0.147
**Gender (M/F)**	57/92	25/30	0.421
**BMI**	26.7 (18.8–42.9)	26.9 (18.4–40.1)	0.788
**THA**			
**Age**	67 (38–97)	65.5 (44–84)	0.166
**Gender (M/F)**	38/48	16/14	0.404
**BMI**	25.1 (18.8–37.2)	27.4 (18.4–32.5)	0.181
**TKA**			
**Age**	71.0 (46.0–86.0)	69.0 (45.0–81.0)	0.402
**Gender (M/F)**	19/44	11/14	0.225
**BMI**	29.7 (20.2–42.9)	26.7 (19.4–40.1)	0.272

Data are reported as median (range). THA, total hip arthroplasty; TKA, total knee arthroplasty; BMI, body mass index; M, males; F, females.

**Table 2:** PROMs in THA patients.

Score	2019	2020	*p* Value (between Groups)
**Preoperative**			
VAS	8 (2–10)	8 (5–10)	0.032
SF-12 MCS	57.875 (16.9–70.4)	54.05 (19.1–69.0)	0.543
SF-12 PCS	30.8 (18.8–59.6)	28.15 (17.7–55.8)	0.146
HOOS-PS	41.7 (20.0–74.8)	48.45 (20.0–100.0)	0.045
HOOS-PS Activity	6 (5–8)	7 (6–8)	0.322
HOOS-PS Function	5 (3–6)	5.5 (4–7.75)	0.049
**3-month follow-up**			
VAS	1 (0–10) ***	1 (0–7) ***	0.602
SF-12 MCS	54.7 (17.0–66.2)	54.9 (22.6–68.7)	0.967
SF-12 PCS	43.8 (20.9–58.5) ***	39.2 (30.0–60.7) ***	0.233
HOOS-PS	21.7 (0.0–61.6) ***	33.9 (0.0–61.6) ***	<0.001
HOOS-PS Activity	4 (3–5) ***	8 (6–8)	<0.001
HOOS-PS Function	1 (0–3) ***	2 (1–4) ***	0.200
Satisfaction	3.0 (0–4)	3.5 (1–4)	0.738

Data are reported as median (range). *** *p* < 0.001 compared to preoperative score in the same group.

**Table 3:** PROMs in TKA patients.

Score	2019	2020	*p* Value (between Groups)
**Preoperative**			
VAS	8 (3–10)	8 (3–10)	0.368
SF-12 MCS	50.5 (20.7–72.0)	53.5 (29.6–72.0)	0.271
SF-12 PCS	30.2 (13.4–49.8)	31.3 (19.4–54.8)	0.617
KOOS-PS	48.5 (27.5–100.0)	48.5 (0.0–72.0)	0.459
KOOS-PS Activity	11 (9.5–12)	12 (11–12)	0.277
KOOS-PS Function	8 (6–10)	7 (6–9)	0.212
**3-month follow-up**			
VAS	4 (0–10) ***	5 (0–10) ***	0.412
SF-12 MCS	55.1 (17.8–65.6)	50.8 (38.8–67.2)	0.624
SF-12 PCS	42.9 (21.0–55.5) ***	40.3 (23.9–55.3) ***	0.778
KOOS-PS	37.0 (0.0–100.0) ***	37.0 (14.8–51.2) ***	0.626
KOOS-PS Activity	8 (6–9) ***	10 (7–12)	0.010
KOOS-PS Function	4 (2–7) ***	2 (0–5) ***	0.039
Satisfaction	2 (0–4)	3 (1–4)	0.078

Data are reported as median (range). *** *p* < 0.001 compared to preoperative score in the same group.

**Table 4:** Items in the HOOS-PS and KOOS-PS and their subscale of origin.

Subscale of Origin	KOOS-PS	HOOS-PS	Component
Activities of Daily Living	Rising from bed	Descending stairs	Functional
	Putting on sock/stockings	Getting in/out of bath	Functional
	Rising from sitting	Rising from sitting	Functional
	Bending on floor		Functional
Sport and Recreational	Twisting on your injured knee	Running	Activity
	Kneeling	Twisting/pivoting on your loaded leg	Activity
	Squatting		Activity

Functional component: subscale F; Activity component: subscale A.

## Data Availability

The datasets used and/or analyzed during the current study are available from the corresponding author upon reasonable request.
